# The Inhibition of CD40/CD154 Costimulatory Signaling in the Prevention of Renal Transplant Rejection in Nonhuman Primates: A Systematic Review and Meta Analysis

**DOI:** 10.3389/fimmu.2022.861471

**Published:** 2022-04-07

**Authors:** Steven Perrin, Marianne Magill

**Affiliations:** ^1^ Eledon Pharmaceuticals, Irvine, CA, United States; ^2^ Marianne Magill, Natick, MA, United States

**Keywords:** CD40, CD40L, CD154, renal transplant, transplant rejection, tolerance

## Abstract

The prevention of allograft transplant rejection by inhibition of the CD40/CD40L costimulatory pathway has been described in several species. We searched pubmed for studies reporting the prevention of kidney transplant rejection in nonhuman primates utilizing either anti CD40 or anti CD40L (CD154) treatment. Inclusion of data required treatment with anti CD40 or anti CD154 as monotherapy treatment arms, full text available, studies conducted in nonhuman primate species, the transplant was renal transplantation, sufficient duration of treatment to assess long term rejection, and the reporting of individual graft survival or survival duration. Eleven publications were included in the study. Rejection free survival was calculated using the Kaplan-Meier (KM) life test methods to estimate the survival functions. The 95% CI for the medians was also calculated. A log-rank test was used to test the equality of the survival curves between control and treatment arms (CD40 and CD154). The hazard ratio for CD154 compared to CD40 and 95% CI was calculated using a Cox proportional-hazards model including treatment as the covariate to assess the magnitude of the treatment effect. Both anti CD40 and anti CD154 treatments prevented acute and long term graft rejection. The median (95% CI) rejection free survival was 131 days (84,169 days) in the anti CD40 treated animals and 352 days (173,710 days) in the anti CD154 treated animals. Median survival in the untreated animals was 6 days. The inhibition of transplant rejection was more durable in the anti CD154 group compared to the anti CD40 group after cessation of treatment. The median (95% CI) rejection free survival after cessation of treatment was 60 days (21,80 days) in the anti CD40 treated animals and 230 days (84,552 days) in the anti CD154 treated animals.

## Introduction

In 2021 more than 40,000 solid organ transplants were performed in the USA, the first time in history this number has been exceeded (UNOS). The steady increase in transplants has been due, in part to the development of calcineurin inhibitors as prophylaxis against cellular and antibody mediated rejection. A major historical innovation in efforts to reduce transplant rejection is the inclusion of the calcineurin inhibitor tacrolimus in standard polypharmacy regimens resulting in reduce cellular and antibody mediated rejection improving short term outcomes for transplant recipients and making transplant a more viable option for patients in need. Although tacrolimus has had a dramatic impact on 1 year survival rates for organ transplant, long term survival rates (> 3 years) have not changed since their introduction, suggesting that further improvements are needed (1).

One approach to improving long term outcomes post-transplantation is through the inhibition of the co-stimulatory pathways of the immune system. These receptors belonging to the immunoglobulin (CD80, CD86, CD28, CTLA4, ICOS, ICOSL, PD-1, PD-L) and TNF (CD40, CD154) superfamilies respectively were first identified as cell surface receptors on immune cells capable of modulating immune cell responses between cells of the T cell lineage and antigen presenting cells (2–11).

The discovery and role of the costimulatory pathway in regulating adaptive and innate immune function more than 30 years ago led to preclinical data in nonhuman primates (NHPs) demonstrating the inhibition of costimulatory signaling *via* CD80 & CD86 (12–16), CD28 & CTLA4 (17–20), and CD40 & CD40L (CD154) (15, 21–32) to prevent acute and long term allograft transplant rejection. Indeed, this led to global approval of belatacept, a CTLA4-FC fusion protein developed by Bristol Myers Squibb for renal transplant indications in 2011. Additional costimulatory antagonists are now in clinical development for renal transplant targeting CD28 (Vel-101, Veloxis), CD40 (Iscalimab, Novartis; ASKP1240, Astellas), and CD154 (AT-1501, Eledon; HZN-4920, Horizon).

Two important hypotheses have arisen from the extensive number of studies conducted in multiple species antagonizing costimulatory receptors to prevent transplant rejection: (1) Antagonizing the CD40/CD154 pathway is more efficacious than the inhibition of other costimulatory pathways; and (2) Inhibition of CD154 is more efficacious than inhibition of CD40 in preventing transplant rejection. In support of this, it was recently reported that anti CD154 was significantly more efficacious in preventing graft rejection compared to anti CD40 therapy in a pig to rhesus macaque xenograft transplant model (33). Indeed, initial clinical development programs focused on inhibition of CD154 due to superior efficacy in preclinical studies. Hu5c8 was a clinical development candidate for tissue transplant and autoimmune disease, but demonstrated unpredicted on target toxicity due to Fc effector function activity and high affinity binding to platelets, resulting in thrombolytic events in humans (34, 35). This halted further clinical development of other anti-CD154 antibodies until solutions to the on target binding could be engineered. Subsequent research suggested the thrombolytic activity of 5c8 is due to binding of 5c8 to CD40L on platelets and is mediated by the FC portion of the heavy chain sequence of 5c8 (36, 37). Furthermore anti CD40L antibodies lacking FC effector function do not activate platelets and do not cause thromboembolisms (26, 38, 39).

CD154 is a costimulatory type II membrane receptor found on activated T helper cells, platelets, endothelial cells, basophils, eosinophils, vascular smooth muscle cells, NK cells, astrocytes, and in some cases on B cells (40–46). The receptor for CD154, CD40 is a transmembrane protein of the Tumor Necrosis Factor Receptor (TNFR) family found on antigen presenting cells (APCs) such as B cells, macrophages, dendritic cells, neutrophils, mesangial cells and tubular cells in the kidney, and microglia in the central nervous system (47–52).

The binding of CD154 to CD40 activates multiple downstream immune and inflammatory responses. Inhibition of CD154 signaling can abolish many effector mechanisms of inflammation with the potential to instill transplant tolerance (53–57) and ameliorate Lupus Nephritis (58), Arthritis (59, 60), Grave’s Disease (61); Multiple Sclerosis (62), and Sjogren’s Syndrome (63). These effects are mediated by inhibition of effector and follicular T cell function, increased T regulatory function, inhibition of germinal center formation, inhibition of B cell maturation and antibody production, and inhibition of antibody class switching (10, 64–72). The inhibition of the CD40/CD154 costimulatory pathway in nonhuman primate models of cellular and organ transplantation has been shown to improve graft function and survival compared to untreated animals. Although there is limited data directly comparing anti CD154 and anti CD40 antibodies in nonhuman primate (NHP) models of renal transplant these studies provide sufficient data for a meta-analysis comparing inhibition of the ligand versus the receptor in preventing transplant rejection as monotherapies.

## Materials and Methods

### Methodology

A systematic review and meta-analysis was performed according to the guidelines that are recommended by the PRISMA statement (Preferred Reporting Items for Systematic reviews and Meta-Analysis).

### Search Terms

A systematic search of peer reviewed articles in Pubmed was conducted between September 2^nd^, 2021 and September 15^th^, 2021. The search term was: “renal transplant AND (CD40 OR CD154)”. Original research studies were included if the study was conducted in rhesus macaques or cynomolgus monkeys, if the transplanted organ was exclusively renal transplant, if the treatment period was at least 90 days, if there were monotherapy treatment arms with antagonistic biologics against either CD154 or CD40, and if survival data was reported for each treated animal.

### Data Extraction

Data was extracted on the species, the name of the protein biologic, the type of protein biologic, the costimulatory target for the biologic treatment, the dosing scheme, the dose of biologic, the duration of dosing, the number of days of rejection free survival post renal transplant, and the number of days each animal survived after cessation of treatment if reported. Individual animal survival data was available for 99 animals and data was estimated based on Kaplan- Meier plots for 3 animals. Animals who did not have rejection or were alive at the end of the trial were censored at the last available time.

### Statistical Analysis

An Individual Participant data meta-analysis was performed to estimate the overall treatment effect on rejection free survival.

Rejection free survival was summarized by the 25^th^, 50^th^ (median), and 75^th^ percentiles calculated using the Kaplan-Meier (KM) life test methods to estimate the survival functions. The 95% CI for the medians was also calculated. A log-rank test was used to test the equality of the survival curves between control and treatment arms (CD40 and CD154). A similar analysis was performed to compare CD40 versus CD154. The hazard ratio for CD154 compared to CD40 and 95% CI was calculated using a Cox proportional-hazards model including treatment as the covariate to assess the magnitude of the treatment effect. KM plots show the survival probabilities over time.

A Cox proportional hazard model, including study treatment was used to assess the impact of covariates on rejection free survival. The covariates examined were species, IgG subfamily and duration of treatment.

An inverse weighting variance random effects meta-analysis model comparing the effect of mean survival time was also examined. An I^2^ measure was used to assess heterogeneity of the data. A forest plot comparing subgroups of CD40 and CD154 is presented with 95% CI for each study and for each combined subgroup.

All statistical analyses were performed using SAS^®^ Version 9.4 or higher and/or the R analysis language (Version 4.0.3 or greater).

## Results

**Figure 1:**
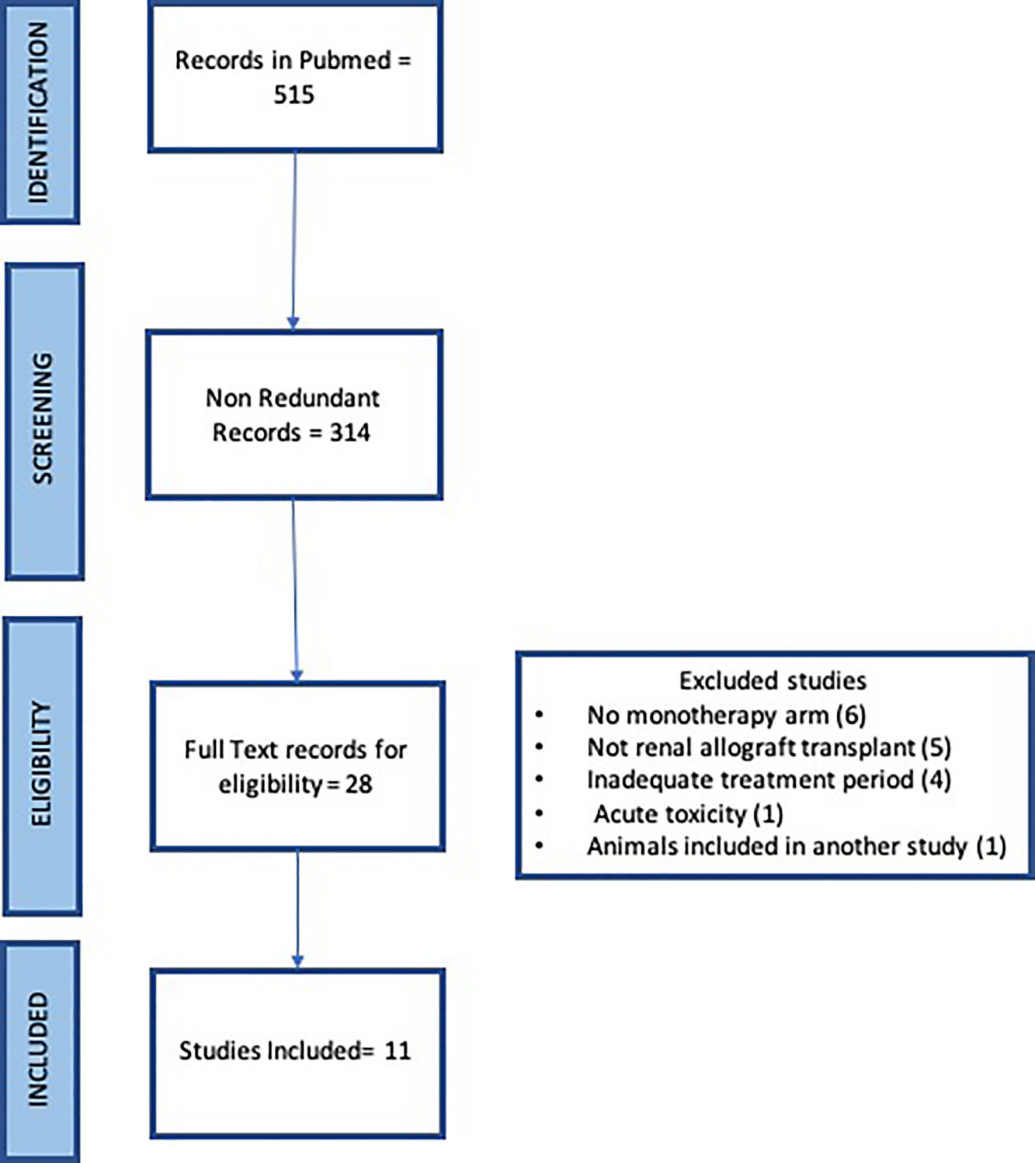
PRISMA Flow Chart of Literature Search.

The systematic search of Pubmed with the term “renal transplant” and “CD154 or CD40” returned 226 and 289 records respectively for a total of 515 records. Redundant records were removed leaving 314 records for manual curation. The titles and abstracts were read for each record to identify original articles testing anti CD154 or anti CD40 antibodies in nonhuman primate studies in either rhesus macaque or cynomolgus monkeys. 28 articles were identified for full text review. Six articles were excluded due to a lack of monotherapy arm, five records were excluded that were not renal allograft, four records were excluded due to brief treatment period to assess rejection, one record saw acute toxicity, and one record reported animals included in another study (**Figure 1**). Eleven articles were identified.

### Characteristics of Curated Data Sets

Eleven full text publications were included in the analysis. These were published between 1999 and 2017, contained dosing information, included a monotherapy arm for an anti CD154 or anti CD40 antibody, and provided individual animal survival data post renal transplant. There were 64 treated animals included in the study, 40 treated with anti CD154 antibodies and 24 treated with anti CD40 antibodies, as well as 38 control or untreated animals. Eight of the studies utilized rhesus macaques (65 animals) and 3 studies utilized cynomolgus monkeys (37 animals). There were 4 different anti CD154 antibodies and 3 different anti CD40 antibodies utilized in the studies. A summary of these data can be seen in **Table 1**.

**Table 1:** Publications included in the study.

Publication	Year	Drug	Target	Dose	Loading Doses	Dosing Interval	Species	# Treated	# Control	Isotype
Kirk et al. (21)	1999	5c8	CD154	20 mg/kg	days pre-op 0, post-op 0, 3, 10, 18, 28	q28d for 5 months	rhesus macaque	9	4	IgG1
Preston et al. (22)	2005	IDEC-131	CD154	20 mg/kg	days -1, 0, 3, 7	q7d for 8 weeks	rhesus macaque	5	5	IgG1
Montgomery et al. (15)	2002	5c8	CD154	20 mg/kg	days pre-op 0, post-op 0, 3, 10, 18, 28	q28d for additional 5 months	rhesus macaque	3	5	IgG1
Xu et al. (23)	2001	5c8	CD154	20 mg/kg	days -1, 0, 3, 10, 18, 28	q28d for 6 months	rhesus macaque	8	5	IgG1
Xu et al. (24)	2002	5c8	CD154	20 mg/kg	days -1, 0, 3, 10, 18, 28	q28d for 12 months	rhesus macaque	6	0	IgG1
Kanmaz et al. (25),	2004	A1793	CD154	20 mg/kg	days 0, 1,4, 11, 18,56,84	Treatment terminated on day 84	rhesus macaque	6	6	IgG1
Kim et al. (26)	2017	CD154 dAB	CD154	30 mg/kg	days 0, 1,4, 11, 18,56,84	Treatment terminated on day 84	rhesus macaque	3	0	dAB
Song et al. (27)	2014	ASKP1240	CD40	5 mg/kg	days -1, 0, 3, 7, 11, 14	2.5 mg/kg q14d until day 168	cynomolgus	6	3	IgG4
Aoyagi et al. (28)	2009	4D11	CD40	20 mg/kg	days pre-op 0, post-op 0,4,11.14	q7d 10 mg/kg for 6 months	cynomolgus	3	3	IgG4
Aoyagi et al. (28)	2009	4D11	CD40	10 mg/kg	days pre-op 0, post-op 0,4,11.14	q7d 5 mg/kg for 6 months	cynomolgus	3	3^1^	IgG4
Imai et al. (29)	2007	4D11	CD40	10 mg/kg	days pre-op 0, post-op 0, 2,4,6,8,10,12.14	q7d for weeks 1-6, q14d for weeks 7-10	cynomolgus	3	3	IgG4
Imai et al. (29)	2007	4D11	CD40	20 mg/kg	days pre-op 0, post-op 0, 2,4,6,8,10,12.14	q7d for weeks 1-6, q14d for weeks 7-10	cynomolgus	3	3^1^	IgG4
Imai et al. (29)	2007	4D11	CD40	20 mg/kg	days pre-op 0, post-op 0, 2,4,6,8,10,12.14	q7d for weeks 1-6, q14d for weeks 7-10	cynomolgus	1	3^1^	IgG4
Haanstra et al. (30)	2003	ch5D12	CD40	20 mg/kg	days -1, 0	10 mg/kg: days 4, 7, 11, and 14; then 5 mg/kg 2q7 until day 56.	rhesus macaque	5	4	IgG1

^1^Same control animals for each treatment group.

### Animal Survival

**Figure 2:**
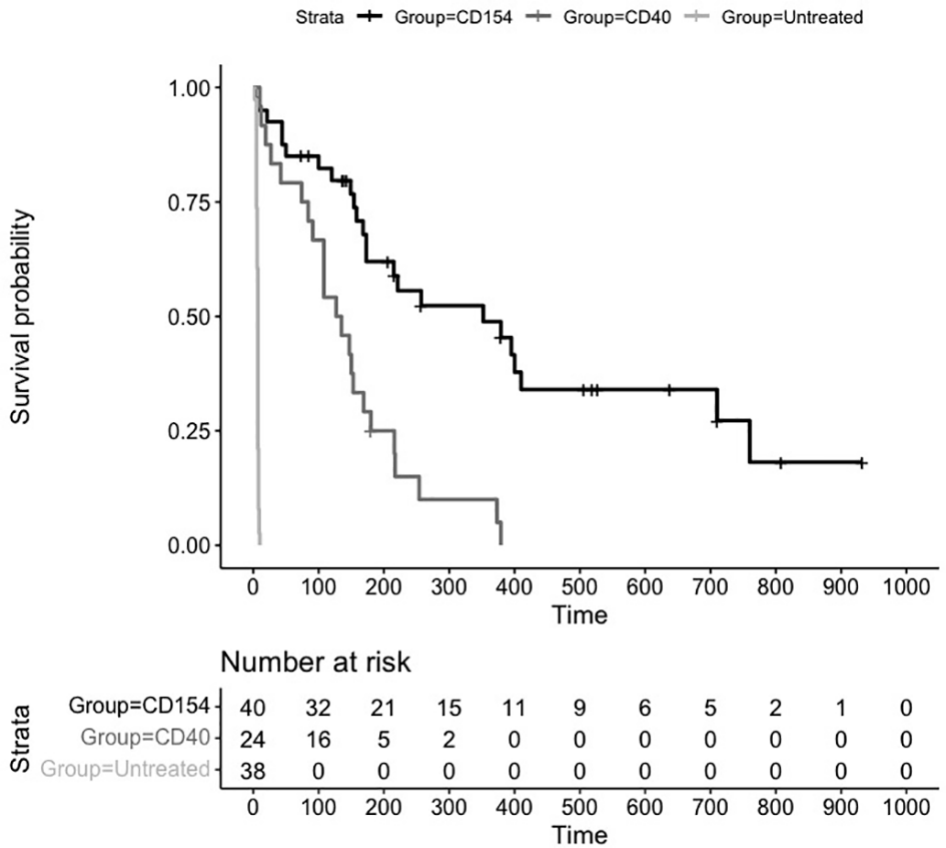
Kaplan Meier Survival Plot and Risk Table for Treatments versus Placebo. Kaplan-Meier estimates of the probability of rejection free survival by treatment group. Median survival untreated (6 days), CD40 (131 days), and CD154 (352 days) groups (P = 0.0001; log-rank test).

An assessment of survival across groups suggested a statistically significant difference between untreated and treated animals as well as between anti CD40 and anti CD154 treated animals. The Kaplan Meier plot and risk table of the survival function are shown in **Figure 2**.

The median (95% CI) rejection free survival was 131 days (84,169 days) in the anti CD40 treated animals and 352 days (173,710 days) in the anti CD154 treated animals. Median survival in the untreated animals was 6 days.

The log rank test comparing the equality of survival curves between anti CD40 and anti CD154 was statistically significant (p<0.0001) indicating an increase in survival for animals treated with anti CD154 compared to anti CD40.

A cox proportional hazard model to assess the treatment variable on the outcome of survival showed the hazard ratio (HR [95% CI]) for anti CD154 compared to anti CD40 was 0.29 [0.15, 0.54] indicating animals treated with anti CD154 have a 71% higher likelihood of survival compared to anti CD40 treated animals (p=0.0001).

### Meta Analysis of Animal Survival With Costimulatory Inhibition

**Figure 3:**
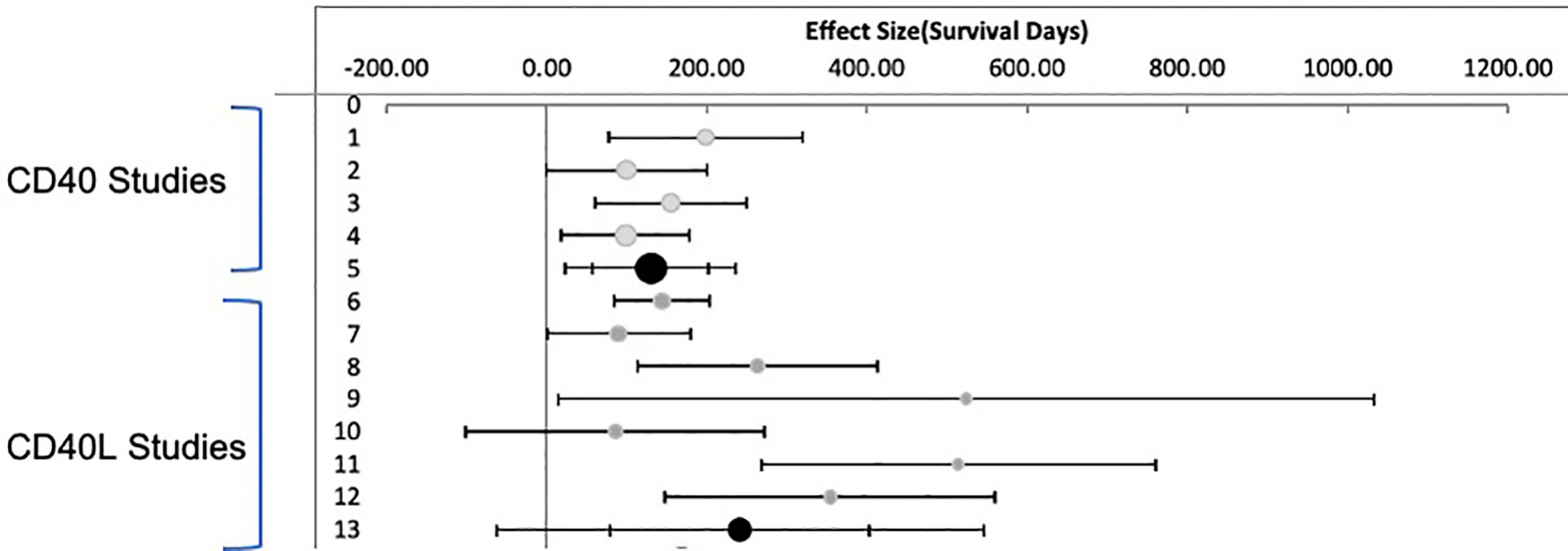
Meta Analysis: Inverse Variance Weighting Random Effects Model. Published studies of anti CD40 and anti CD154 antibodies in nonhuman primates. Forest plot displaying random effects meta-analysis results of the association compared to nontreated animals. The Black circle is the mean values for treatment effect across all studies. Light Grey circles are anti CD40. Dark Grey circles are anti CD154.

Random effects meta- analysis demonstrated that monotherapy costimulatory inhibition was effective at preventing renal transplant rejection in nonhuman primates (combined effect size 183.7 days: 95% CI, 95.1 to 272.3 days) p <= 0.001. There was, however, a high degree of heterogeneity across the studies (I^2^ = 78%) reflecting a difference in effect size among subgroups. A subgroup analysis of anti CD40 versus anti CD154 demonstrates that anti CD154 had a higher effect size 241.4 days (95% CI, 79.6, 403.2 days) compared to anti CD40, 130.1 days (95% CI, 57.7 to 202.5 days) as shown in **Figure 3**. The model does indicate that the studies included for the anti CD40 studies are much more homogeneous than the anti CD154 (I^2^ = 30.1% vs. 85.2% for the anti CD40 and anti CD154 subgroups respectively).

### Duration of Treatment Influences Time to Transplant Rejection and Survival

An analysis was performed to assess whether there were any covariates associated with the difference in survival times between anti CD154 and CD40 treated animals. There were significant differences between anti CD154 and CD40 groups with regards to species, the IgG subfamily of the antibodies, as well as duration of treatment. The majority of studies using anti CD154 antibodies were conducted in rhesus macaques (all 7 studies) compared to anti CD40 studies where six studies were conducted in cynomolgus monkeys and one was conducted in rhesus macaques. Similarly, all four of the anti CD154 antibodies were IgG1 subclass (one is an IgG1 domain antibody) whereas one of the anti CD40 antibodies was IgG1 and 2 were IgG4. Given there were no studies using anti CD154 antibodies in cynomolgus monkeys and none of the anti CD154 antibodies were IgG4, it is impossible to rule out difference in survival with these two variables.

The studies consisted of an induction phase of treatment when antibodies were administered every few days for up to fourteen days. The induction was then followed by maintenance therapy for a variable number of days between studies with varying doing frequencies. We analyzed the survival function with a cox proportional hazard model including the duration of exposure to either anti CD40 or anti CD154 antibodies to assess whether duration of treatment accounted for the differences observed in survival times between anti CD154 and anti CD40 studies. Time of exposure is a significant predictor in graft function and survival (p<0.001). The hazard ratio (HR[95% CI]) for CD154 compared to CD40 controlling for exposure is 0.46 (0.24, 0.90) indicating that inhibition of CD154 was still superior to inhibition of CD40 in preventing graft rejection and increasing survival, suggesting that animals on anti CD154 are 54% more likely to survive even with longer exposure of inhibiting either CD40 or CD154.

### Costimulatory Treatment Induces Long Term Transplant Tolerance

**Figure 4:**
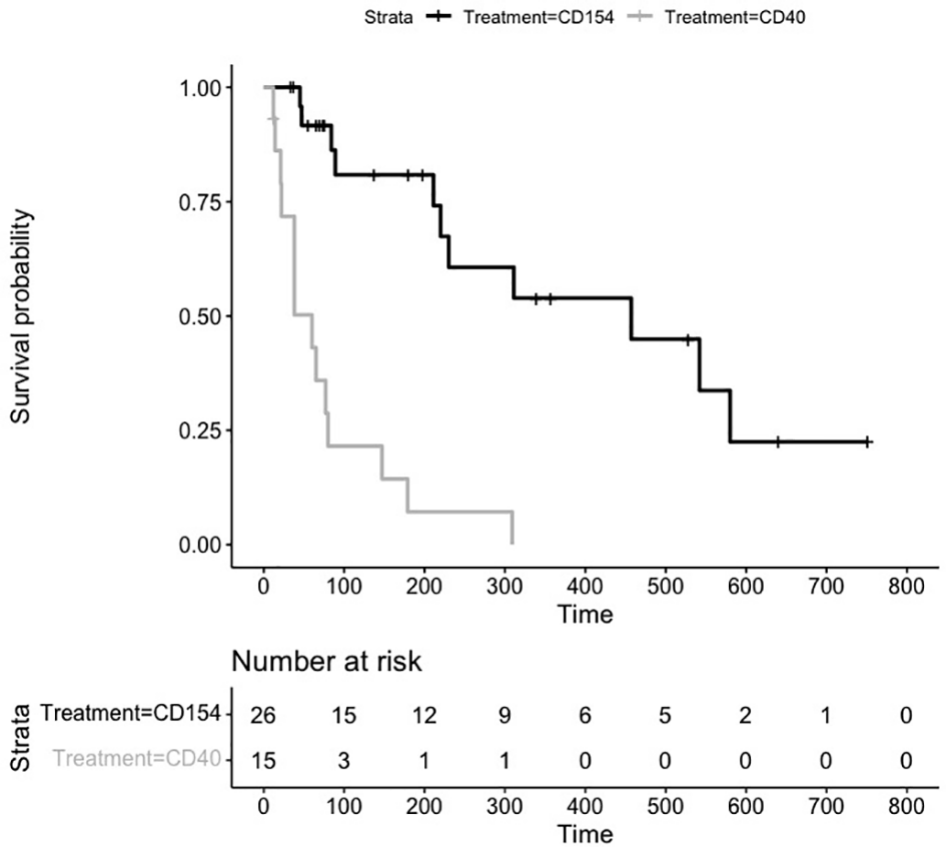
Kaplan Meier Survival Plot and Risk Table for Treatment Durability. Kaplan-Meier estimates of the probability of rejection free survival by treatment group after discontinuation of treatment. Median survival CD40 (38 days), and CD154 (198 days) groups (P = 0.0003 log-rank test).

For many of the studies, treatment was stopped and the durability of preventing transplant rejection was assessed by the number of days without treatment until graft failure. As can be seen from the untreated animals (**Figure 2**), graft rejection occurs very quickly in the absence of immunosuppressive or immunomodulatory treatment (median survival in control animals 6 days). The induction of transplant tolerance has been described in the context of blocking costimulatory signaling as well as in the induction of chimerism with whole body irradiation and bone marrow transplant. There were 26 animals in the anti CD154 cohort and 15 animals in the anti CD40 cohort that were followed for durability of treatment effect. The Kaplan Meier plot and risk table are shown in **Figure 4**.

The median (95% CI) rejection free survival was 60 days (21,80 days) in the anti CD40 treated animals and 230 days (84,552 days) in the anti CD154 treated animals.

The log rank test comparing the equality of survival curves between anti CD40 and anti CD154 was statistically significant (p<.0001) indicating an increase in survival for animals treated with anti CD154 compared to anti CD40.

A cox proportional hazard model to assess the treatment variable on the outcome of survival showed the hazard ratio (HR [95% CI]) for anti CD154 compared to anti CD40 was 0.21 [0.097, 0.475] indicating animals treated with anti CD154 have a 79% higher likelihood of survival compared to anti CD40 treated animals (p=.0002).

### Histopathology

The histopathology data across the eleven articles is sparse and limited in consistent pathological assessments of just the monotherapy arms in the studies. In general, for anti CD154 treated animals with protocol biopsies at specific time points in the absence of overt signs of renal failure there is no histological signs of renal damage and minimal perivascular immune cell infiltrate and no infiltrate present in tubules or glomeruli (21, 24–26) With treatment withdrawal rejection occurred and immune cell infiltrates were associated with tubulitis, endothelitis, and eventually severe damage of the kidney parenchyma (24, 25). The majority of anti CD40 treated animals also had minimal perivascular immune cell infiltrate and no infiltrate present in tubules or glomeruli. With the 4d11 anti CD40 antibody, however there were animals still on treatment that showed mild interstitial cell infiltrate. In addition one animal had tubulitis and severe interstitial fibrosis and tubular atrophy in another animal (28). Imai reported similar results at 1 month all animals having mild interstitial cell infiltration, with or without slight tubulitis and 2 animals having moderate to severe interstitial infiltration along with moderate tubular changes (29). Animals remained stable after 1 month with no changes in pathology until cessation of treatment. After cessation of treatment animals had moderate to severe interstitial fibrosis with a mild tubular atrophy, and mild arteritis without intimal thickening and glomerulopathy was seen in three animals (29).

## Discussion

This meta-analysis of eleven peer reviewed publications contained time to event information regarding renal graft function and renal graft survival data for 64 monotherapy treated animals as well as 38 untreated control animals. While inhibition of either CD40 or CD154 produced a significant advantage in rejection-free survival compared to untreated controls, there was a significant decrease in risk of graft rejection and improvement in survival for animals treated with anti CD154 antibodies compared to anti CD40 antibodies. There was also a significant improvement in graft function and survival influenced by duration of treatment. The histological data also suggested more limited immune cell infiltrate and pathological changes in anti CD154 treated animals compared to anti CD40 treated animals. An interesting observation arising from the analysis is the induction of transplant tolerance and durability of preventing graft dysfunction and rejection after cessation of treatment with anti CD154 antibodies having longer durability of effect than anti CD40 antibodies.

There are three general pathophysiological mechanisms leading to transplant rejection, hyperacute rejection occurring in minutes or hours due to preformed anti donor antibodies, acute rejection occurring in weeks to months of transplant which can be cellular or humoral in nature, and chronic allograft rejection which develops over a period of months to years and is described as transplant vasculopathy (TV) (73), characterized by neointima formation. Hyperacute rejection is uncommon due to advances in prescreening for anti-donor antibodies. Acute and chronic graft rejection are characterized by both vascular and parenchymal damage to the graft involving professional antigen presenting cells as well as “semiprofessional” antigen presenting cells such as platelets and endothelial cells (EC). EC are a natural barrier between graft and host immune systems but can be compromised by pro-inflammatory activation, ischemic reperfusion injury or infections. ECs when activated release inflammatory mediators and can directly activate platelets, NK cells, and T cells.

Significant progress has been made in elucidating the mechanisms by which CD154 inhibition prevents acute and long-term transplant inhibition and, in some cases, can induce transplant tolerance. Accumulating evidence suggest that CD40/CD154 signaling is dynamic in nature, evolutionarily designed to be potent in the recognition of and requirement for immune system activation yet transient to prevent long lasting autoimmune activation. In addition, the focal expression of this receptor/ligand pair on endothelial cells, vascular smooth muscle cells and platelets in the context of atherosclerosis and vasculopathy perfectly juxtapositions the regulation of CD40/CD154 signaling in immune responses to foreign pathogens and “non-self’ in the context of transplant rejection and autoimmunity as well as the site where these events primarily occur in transplant rejection. In addition, in renal allograft transplantation there is the additional relevance to the expression of CD40/CD154 on immune cell infiltrates, tubules, mesangial cells, and glomerular structures in the kidney in allograft transplant and autoimmune nephritis.

There are clear distinctions differentiating between CD154 and CD40 as drug targets and in how they modulate immune responses that may account for these observations. CD154 is a costimulatory type II membrane receptor initially characterized on activated T helper cells. Cell surface CD154 is not constitutively found on the cell, it is inducible and is regulated by T cell receptor and CD28 signaling on the cell surface (74). CD154 is stored intracellularly in microsomes prior to activation as a monomer and trimerizes within seconds to minutes on the cell surface as a functional receptor (41, 75, 76). The activity of CD154 on the cell surface of T cells and platelets is modulated by metalloproteases and the protease ADMA-10 cleaving CD154 on the cell surface and releasing biologically active soluble CD154 (sCD154) (77, 78). The shedding of cell surface CD154 is thus a feedback mechanism which controls constitutive activation of its receptors on APCs. Soluble CD154 is a potent chemokine that then binds to CD40 as well as α5β1 integrin on APCs modulating pro-inflammatory signaling (76, 79). The activity of activated T cells is further modulated by CD154 and sCD154 in a cis fashion by binding with α5β1 integrin on the surface of T cells and inhibiting apoptosis (80) Indeed, T lymphocytes in autoimmune diseases express higher levels of sCD154 and express aberrant amounts α5β1 integrin on the cell surface which increases T cell populations *via* inhibition of apoptotic signaling (81, 82).

Cell surface CD154 on activated T cells interacts with its constitutively expressed receptor, CD40, found on cells of the monocyte lineage and antigen presenting cells (APCs) including B cells (83, 84). B cell maturation, class switching and proliferation requires both antigen and CD40 ligation *via* CD154 on the cell surface of activated T cells because activated T cell supernatants are insufficient to induce full B cell maturation (85). Further evidence supporting the critical role for CD154 in B cell maturation and class switching arises from persons living with X chromosome-linked hyper-IgM Syndrome (HIGM1). HIGM1 arises from mutations in the CD154 gene on the X chromosome and is characterized by undetectable serum levels of IgG, IgA, and IgE with normal or elevated levels of IgM (64). In addition people with HIGM1 either lack or have poorly differentiated germinal centers in secondary lymphoid tissues. Germinal centers are formed in secondary lymphoid tissues during the process of immune responses to antigen presentation by dendritic cells to immature B cells and the maturation of B cells *via* interaction with activated T cells. The expression of CD40 on dendritic cells and B cells and the expression of CD154 on activated T cells in the germinal center is required for B cell maturation, class switching, antibody production and the formation of both memory B cells and long-lived plasma cells (10, 64–69).

The inhibition of CD40/CD40L signaling on T cells, APCs, endothelial cells (ECs), platelets and other non-professional antigen presenting cells is a potent mechanism to inhibit acute and chronic transplant rejection *via* the inhibition of adaptive and humoral immunity induced between donor and recipient immune systems and to modulate the progression of transplant vasculopathy (TV), the chronic process mediating chronic rejection.

The inhibition of CD154 results in decreased infiltration and activation of alloreactive CD4^+^ and CD8^+^ T cells in allograft transplants. The binding of CD154 to CD40 and the integrin α5β1 on CD8^+^ and CD4^+^ T cells respectively results in proliferation and pro-inflammatory signaling on T cells. Anti CD154 antibodies interfere with these interactions resulting in T cell anergy and apoptosis (70, 71, 80, 86). CD40^+^ cellular infiltrates have been characterized in human kidney transplants with chronic rejection. CD40^+^ infiltrates are primarily T cell receptor positive T lymphocytes (73%) and 27% CD68^+^ macrophages (87). CD154 expression was observed on glomerular endothelial, mesangial, and epithelial cells in biopsies from subjects undergoing chronic rejection whereas no CD154 staining is detected in non rejecting biopsies (87). CD40^+^ infiltrates colocalize with CD154 expression in glomeruli and tubules suggesting a role in mediating pro-inflammatory signaling in the kidney and as discussed a role in the development of vasculopathy observed during chronic rejection.

Inhibition of CD40/CD154 induces anergy in peripheral high affinity alloreactive CD4^+^ and CD8^+^ T cells which can be enhanced in the presence of donor specific transfusion of recipient cell populations. The potency of CD154 inhibition and alloreactive T cell anergy is a result of the inhibition of costimulatory signals on antigen presenting cells as well as on alloreactive T cells (55, 86, 88). In a similar fashion, the engagement of CD154 with both CD40 and the integrin CD11b, a component of the MAC complex on dendritic cells and APCs results in secretion of pro-inflammatory cytokines, processes that are inhibited by anti CD154 antibodies (89).

A unique and critical component of CD154 inhibition is the polarization of allogenic T cells (CD4^+^ lymphocytes) that do not undergo anergy and apoptosis to become tolerogenic FoxP3^+^ T regulatory cells (Tregs) (70–72). Blocking CD154 signaling on Tregs results in the proliferation of CD4^+^CD25^+^ (Tregs) which create a tolerogenic environment *via* inhibition of IL2 production (90–92). The Inhibition of IL2 production by Tregs suppresses expansion of donor derived allogenic T cells and is required for the prevention of transplant rejection (93).

The inhibition of CD40/CD154 may also impact chronic rejection by ameliorating EC and platelet activation decreasing transplant vasculopathy (TV). TV is characterized by lesions consisting of a progressive narrowing of the vessel lumen due to intimal hyperplasia. The thickened intima is formed by endothelial infiltrates of host-derived T cells and macrophages and subsequent proliferation of graft-derived SMC and associated extracellular matrix.

Endothelium is the primary interface between donor and recipient immune systems in solid organ transplant. The activation of EC, platelets and leukocyte migration play a critical role in vascular injury. Loss of integrity of the endothelium and activation of ECs balance cellular and antibody mediated transplant rejection. Disruption of the endothelial cell layer results in EC and platelet activation as well as migration of neutrophils, monocytes, and T cells *via* thrombin receptors. In addition ligation of anti HLA antibodies on the surface on the endothelium results in EC activation and expression of adhesion molecules and additional pro-inflammatory cytokines including intercellular cell adhesion molecule-1 (ICAM-1), vascular adhesion molecule-1 (VCAM-1), monocyte chemoattractant protein-1 (MCP-1) and CD40 resulting in adhesion of leukocytes *via* Fcγ receptors (73, 94–96). Platelets are the most abundant source of CD154. Expression of CD40 on ECs results in binding to cell surface CD154 on platelets resulting in the secretion of potent chemoattractants including CXCL4, CXCL7, RANTES, and MCP-1 thus arresting inflammatory T cells, monocytes, and neutrophils at the site of platelet activation (97, 98). Activated infiltrating T cell expressing CD40L interact with CD40 on the surface of platelets further activating their pro-inflammatory potential (99, 100). Although CD40 is also expressed on the cell surface of platelets unlike its expression on other cell types there is no evidence for a direct involvement of CD40 in the activation or aggregation of platelets. Much like α5β1 controls CD154 pro-inflammatory activity by cis acting interactions on T cells, CD40 interactions with CD154 in cis on platelets result in internalization and cleavage of sCD154, a central mechanism controlling CD154 pro-inflammatory activities again showing a negative feedback of CD154 activity (13). In animals models of vascular injury and restenosis, inhibition of CD154 reduces neonatal intima formation and macrophage infiltration (101). These effects are mediated through multiple pathways including P-selectin on platelets and MAC-1 on leukocytes (101, 102). sCD40L stimulates Mac-1 integrin expression and neutrophils adhesion to activated platelets and these activities can be abrogated *via* anti CD154 antibodies (101).

In addition eosinophils express CD154 and the number of eosinophils in circulation are a biomarker of solid organ transplant rejection (44, 103). Activated Basophils express CD154, IL4 and IL13 and induce B cell expression of IgE (43). IgE antibodies specific for donor MHC class I and MHC class II antigens developed during graft rejection in several mouse transplantation models and resembled DSAs associated with vasculopathy and ABMR (104).

Both anti CD154 and anti CD40 antibodies have progressed into clinical development programs in autoimmunity and solid organ transplant. Iscalimab (CFZ-533), an IgG1 anti CD40 antibody reported positive data in a phase 2 study of iscalimab versus a standard of care (SOC) arm containing the calcineurin inhibitor tacrolimus (TAC) in *de novo* kidney transplant recipients. At the 6 month composite endpoint of biopsy proven acute rejection (BPAR), graft, loss or death, iscalimab in conjunction with corticosteroids (CS) and mycophenolate mofetil (MMF) showed non inferiority to CS, MMF, and TAC (21.2% versus 22.2%). The iscalimab group also demonstrated improved renal function (55.8 mL/min versus 45.5 mL/min), fewer serious adverse events (47.1% versus 61.1%), fewer opportunistic infections, and a decreased incidence in rate of new onset diabetes mellitus after transplantation (NODAT) (14.7% versus 38.9%) (105). In a small subgroup analysis 12 subjects in the study (5 in the iscalimab arm and 7 in the TAC arm) had extended treatment to 24 months and underwent protocol biopsies. Biopsies were reviewed with Banff criteria and the chronic allograft damage index (CADI) was calculated. Three of five subjects in iscalimub group had normal renal histology versus none of the subjects in the SOC group. The average CADI score in the iscalimab group was 1.6 +/- 0.6 and 5.1 +/- 0.8 in the SOC group (106). These encouraging results led to further development of iscalimab with the initiation of a phase 2B study in 420 subjects undergoing a *de novo* renal transplant (NCT03663335). The study was discontinued in 2021 after an interim analysis suggested iscalimab was less efficacious than TAC in the prevention of kidney transplant rejection. A clinical study of iscalimab in liver transplant rejection is still ongoing (NCT03781414).

Bleselumab (ASKP1240) an IgG4 anti CD40 antibody completed a phase 2 study in subjects undergoing *de novo* renal transplant. Two study arms with bleselumab (bleselumab plus MMF or bleselumab plus TAC) were compared to a SOC regiment containing TAC. Only 62.4% of subjects achieved the 6 months endpoint with the majority of drop outs in the Bleselumab groups due to adverse events (33/35, 94.2%) (107). The Bleselumab plus TAC group was noninferior to SOC at the 6 month endpoint for BPAR (9.1% versus 6.3% respectively) but the Bleselumab plus MMF did not achieve noninferiority to SOC (37%). There was no statistically significant difference between the SOC group and either of the Bleselumab groups for patient or graft survival at 6 or 36 months (107). The incidence of infections and NODAT was similar between the groups.

VIB4920 an anti CD154 TN3 fusion protein is in a phase 1 study in combination with Belatacept in the prevention of transplant rejection for subjects undergoing *de novo* kidney transplantation, but study results have not been reported at this time (NCT04046549).

The induction of transplant tolerance is considered the “Holy Grail,” or penultimate goal, for the prevention of acute and long term transplant rejection. Initial studies in nonhuman primates identified limitations utilizing whole body irradiation or lymphoid irradiation in conjunction with CD34+ cells to induce chimerism and tolerance due to incomplete T cell depletion in secondary lymphoid organs (108, 109). The addition of CTLA4 costimulatory inhibition with belatacept significantly improved long term graft rejection (110). However, anti CD40L inhibition was shown to be more effective at inducing long term transplant tolerance (111).

For several of the studies analyzed in this meta- analysis, treatment was terminated at specified times due to limitations in antibody availability, space, and costs associated with long term animal care. The half-life of IgG1 and IgG4 antibodies range from nine to eighteen days in nonhuman primates. Animal survival was tracked after cessation of treatment until graft failure showing long term graft survival in the absence of immunosuppression. Animals in the anti CD154 treatment groups had a 3 fold higher likelihood of survival compared to anti CD40 treated animals.

Although this meta- analysis demonstrated a significant difference between the inhibition of CD40 and CD154 in preventing transplant rejection and inducing transplant tolerance after cessation of treatment, the study has several limitations. The strength of the analysis is the availability of individual animal data from multiple studies. Although there were individual animal data for over 100 animals included in the study the actual number of animals per study was small. One study was excluded from analysis utilizing the anti CD154 antibody ABI793 in the prevention of kidney transplant rejection in cynomolgus monkeys due to acute tubular necrosis in the kidneys (ATN). ATN appeared in 8 of 9 animals in the study but the mechanism for the acute toxicity is unclear given that ABI1793 was studied in a renal transplant study in rhesus macaques with no evidence of ATN (25). There were also differences in the species and type of FC utilized with anti CD40 studies being comprised of studies conducted in cynomolgus animals with mostly IgG4 containing FC antibodies and anti CD154 studies being conducted in rhesus macaques with IgG1 containing FC antibodies. Studies have shown similar binding affinities of CD154 antibodies to multiple nonhuman primate species (baboon, rhesus macaques, cynomolgus) however this variable could not be controlled for in this analysis. There was some evidence to suggest pathological differences between anti CD154 and anti CD40 treatment but these interpretations are limited given the sparsity of the data and histopathology was not assessed in a blinded fashion with a single pathologist. Most importantly there were no studies that directly compared the inhibition of anti CD40 versus anti CD154 antibodies in the context of preventing transplant rejection or the induction of transplant tolerance.

In conclusion, this meta-analysis of published studies utilizing anti CD154 and anti CD40 antibodies as monotherapy demonstrates that they are effective at the prevention of acute and long term transplant rejection. These analyses also suggest the ability to induce transplant tolerance after blocking the CD40/CD154 costimulatory pathway in nonhuman primates. Animals treated with anti CD154 had an approximately 3 fold higher likelihood of survival and induction of tolerance compared to anti CD40 treated animals. In addition, the ability to prevent transplant rejection after cessation of treatment was more durable in the anti CD154 treatment groups. Further studies will be required in combination with other technologies to assess the ability to sustain long term transplant tolerance in nonhuman primates.

## Data Availability Statement

The datasets presented in this study can be found in online repositories. The names of the repository/repositories and accession number(s) can be found in the article.

## Author Contributions

Initial idea: SP. Data collection and analysis: SP and MM. Data interpretation: SP and MM. Writing - first draft: SP. Writing - Review and Editing: SP and MM. All authors contributed to the article and approved the submitted version.

## Conflict of Interest

SP declares that he is employed by Eledon Pharmaceuticals. Eledon is developing an anti CD40L antibody for renal and islet cell transplant. MM is an independent paid statistical consultant to Eledon Pharmaceuticals.

The remaining authors declare that the research was conducted in the absence of any commercial or financial relationships that could be construed as a potential conflict of interest.

## Publisher’s Note

All claims expressed in this article are solely those of the authors and do not necessarily represent those of their affiliated organizations, or those of the publisher, the editors and the reviewers. Any product that may be evaluated in this article, or claim that may be made by its manufacturer, is not guaranteed or endorsed by the publisher.
